# Metagenomic analysis of microbial community associated with coral mucus from the Gulf of Aqaba

**DOI:** 10.1016/j.heliyon.2019.e02876

**Published:** 2019-11-28

**Authors:** Emad Hussien, Abdul-Salam Juhmani, Ruba AlMasri, Fuad Al-Horani, Mohannad Al-Saghir

**Affiliations:** a Department of Biological Sciences, Yarmouk University, Irbid, Jordan; b Department of Food Science and Human Nutrition College of Applied and Health Sciences, A'Sharqiyah University, Ibra, Oman; c Department of Environmental Sciences, Informatics and Statistic, Ca’ Foscari University of Venice, Venice, Italy; d Department of Marine Biology, The University of Jordan, Aqaba, Jordan; e Department of Biological Sciences, Ohio University, Zanesville, OH, 43701, USA

**Keywords:** Microbiology, Coral health, Coral damage, Coral, Jordanian coast, Red sea coral, Pyrosequencing, Marine science station, Gulf of Aqaba, Microbial community shift

## Abstract

Coral-associated microbial communities contribute to a wide variety of useful roles regarding the their host, and therefore, the arrangement of the general microbiome network can emphatically impact coral wellbeing and survival. Various pollution sources can interfere and disrupt the microbial relationship with corals. Here, we adopted the bacterial tag-encoded FLX amplicon pyrosequencing (bTEFAP®) technique to investigate the shift of microbial communities associated with the mucus of the coral *Stylophora pistillata* collected from five sites (Marine Science Station, Industrial Complex, Oil Terminal, Public Beach, and Phosphate Port) along the Gulf of Aqaba (Red Sea). Our results revealed a high diversity in bacterial populations associated with coral mucus. Proteobacteria were observed to be the dominating phylum among all sampling sites. The identified bacterial taxa belong to the pathogenic bacteria from the genus *Vibrio* was presented in varying abundances at all sampling sites. Diversity and similarity analysis of microbial communists based on rarefaction curve and UniFrac cluster respectively demonstrated that there are variances in microbial groups associated with coral mucus along sites. The pollution sources among different locations along the Gulf of Aqaba seem to affect the coral-associated holobiont leading to changes in bacterial populations due to increasing human activities.

## 1 Introduction

Corals are diverse meta-organisms that provide an essential bio-habitat for many other marine species (Mulhall, 2009), for instance, bacteria, Archaea, and microalgae (zooxanthellae) (Rosenberg et al., 2007). Unfortunately, recent research indicates that more than 30% of coral reefs have been destroyed due to emerging diseases (Harvell et al., 2007). Some of these diseases are attributed to coral-pathogenic microorganisms and other factors (Rosenberg and Ben-Haim, 2002; Hughes et al., 2003).

The microorganisms that are associated with reef–building, which is also called coral holobiont, have been widely investigated because of a real influence on coral physiology and well-being (Carlos et al., 2013). Microorganisms linked to corals appear to strengthen the host's well-being by supplying a nutritional source and protecting the host from other pathogenic bacteria by the production of antimicrobial compounds (Ritchie, 2006). These microbial communities exist naturally in various anatomic sites in the coral including the surface mucus layer and the coral tissues (Bourne and Munn, 2005). Coral mucus contains various microorganisms that present benefits to their host by different means, including photosynthesis, nitrogen fixation, the delivery of nutrients, and inhibition of sickness (Rosenberg et al., 2007). Moreover, the coral-microbial association is distinct from the surrounding habitat, containing species that are different from the free-living seawater microbes (Rohwer et al., 2001; Carlos et al., 2013). Recently, many efforts have focused on characterizing these microbial communities and their specific metabolic role in coral health (Lesser et al., 2004; Olson et al., 2009; Zhang et al., 2015; Zaneveld et al., 2016; Welsh et al., 2016).

The shift of the structure and diversity of associated microbial communities could play a key role in adapting corals to rapid environmental changes (Reshef et al., 2006). Recently, local and worldwide, ecological disturbances have intensely compromised the well-being of corals, which has thus influenced the entire biological community of the coral reef (Harvell et al., 2007; Wilkinson, 1999; Green and Bruckner, 2000; Gardner et al., 2003; Pandolfi et al., 2003; Lesser et al., 2007). Recent reports show that 58–70% of coral reefs globally are endangered due to human activities (Hughes et al., 2003; Weil et al., 2006).

Different environmental stressors endanger the coral reef ecosystem in the Gulf of Aqaba (GOA). Many pollution sources threaten the Jordanian coast of GOA, including urban expansion, tourist developments, oil leakage, phosphate dust spillage during loading of phosphate minerals, and industrial and solid waste discharge (Al-Horani et al., 2006). Consequently, these factors can shift the diversity of the coral-associated microbial communities which consequently affect the role of coral protection against pathogens.

Coral mucus functions are essential to the coral, including providing a defense mechanism against environmental stresses (temperature and salinity in the coral surrounding water), in ciliary-mucus feeding, in sediment cleansing, a shield from UV radiation, and regulating surface bacterial growth (Brown and Bythell, 2005). Different studies were conducted on the coral associated microbial communities in the GOA area using culture-based techniques for the identification and characterization of bacteria. For example, Lampert et al. (2006) characterized cultivable bacteria within the mucus of healthy Red Sea solitary coral *Fungia scutaria*. Jaber (2012) have also studied the mucus related bacterial communities of *Stylophora pistallata* and *Galaxea fascicularis* collected from the Jordanian coast of the GOA during different seasons. Furthermore, Telfah (2012) studied the differences in the microbial communities associated with *Pocillopora damicornis* and *S. pistillata* growing at various contaminated and uncontaminated sites in the Jordanian coast. In recent research, the bacterial communities associated with soft coral in the Red Sea were studied, and the dominant bacterial species which displayed different antimicrobial activities against various pathogens were characterized (ElAhwany et al., 2015).

The recent rapid advances in DNA sequencing technologies have given vital bits of knowledge into patterns of microbial diversity and function of coral reefs, improving our perception of how microorganisms impact the overall function and stability of the coral reef ecosystem (Hussein et al., 2017; Meyer et al., 2014). Indeed, these technologies provide opportunities to explore these complex microbial associations and their response mechanisms to environmental stresses (Kysela et al., 2005).

In this study, we assessed the bacterial community structure associated with the mucus of the hard-coral *S. pistillata* inhabiting sites influenced by different sources of pollution in the GOA. Bacterial community structure was characterized using Roche GS FLX Titanium technology based on 454-pyrosequencing. Furthermore, we investigated the harmful bacterial species that affect coral health to highlight the health status of coral in the GOA.

## 2 Materials and methods

### 2.1 Study area

**Fig. 1:**
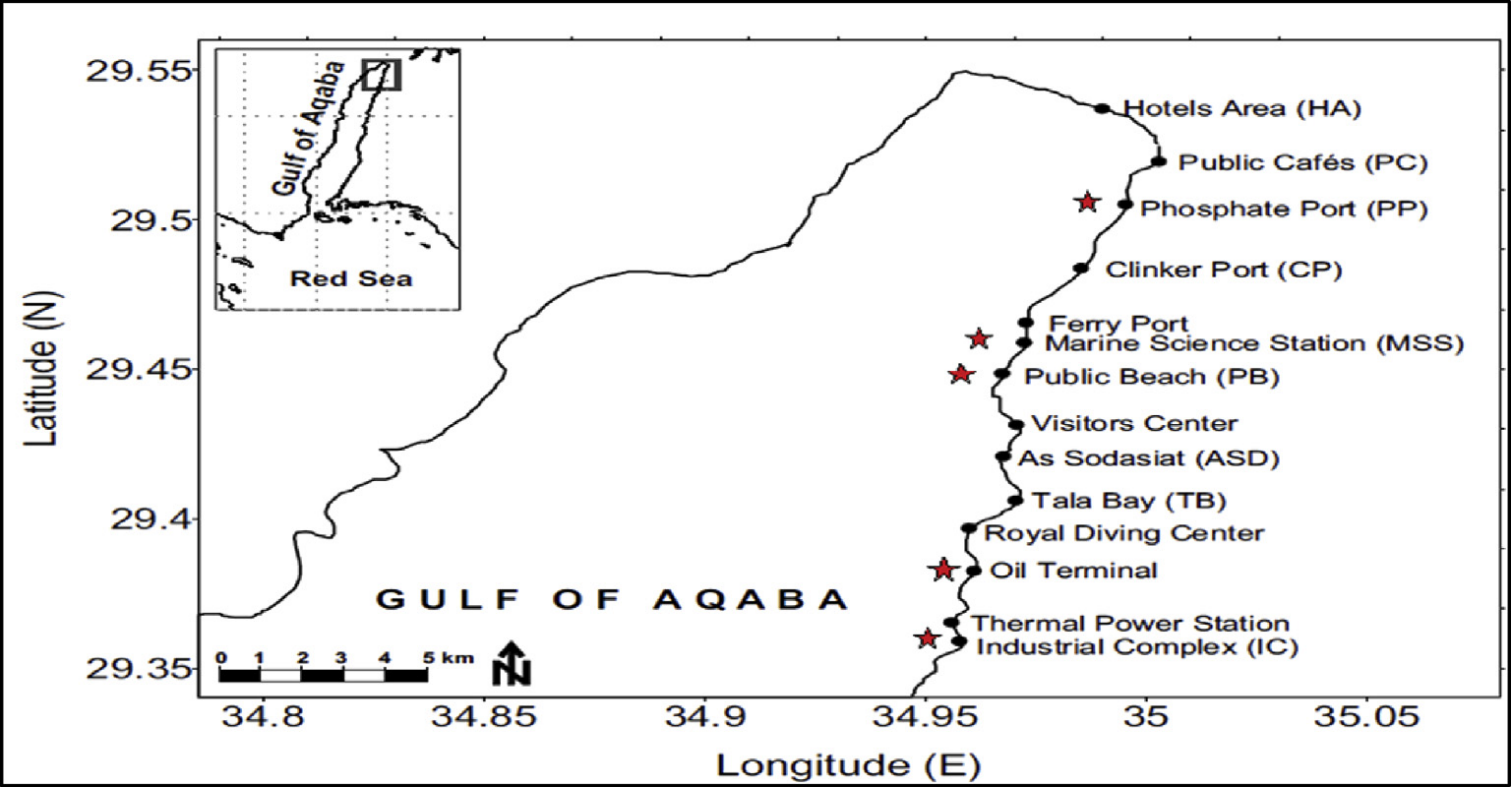
Map of the GOA showing the sampling sites along the Jordanian coast of the Red Sea. Red stars indicated the collection sites.

This study was carried out in the coral reef located in the Jordanian coast of GOA, the northern-eastern extension of the Red Sea (Fig. 1). The samples were collected at five different sites; 4 sites threatened by different sources of pollution (AQ2: Oil Terminal, AQ3: Industrial Complex, AQ4: Public Beach and AQ5: Phosphate Port) and one control uncontaminated site (AQ1: Marine Science Station) (Table1). The Red Sea coral reef status has fluctuated since 2002 (Wilkinson, 2004). Coral damage was reported in the GOA; because of the increased rate of coastal enhancement and increased level of pollutants (Al-Horani et al., 2006). The status of selected sites was determined based on previous literature as illustrated in Table 1.

**Table 1:** Sampling sites in the GOA and the type of pollution.

Site	Location	Major Source of pollution	Reference
AQ1	Marine Science Station	Uncontaminated site	Wahsha et al. (2017)
AQ2	Oil Terminal	Oil transportation, oil spills	Al-Rousan et al. (2005)
AQ3	Industrial Complex	Phosphate fertilizer, potash export terminal, mixed fertilizer plant	Al-Horani et al. (2006)
AQ4	Public Beach	Engine exhaust gas emission, solid waste discharges	Al-Absi et al. (2016)
AQ5	Phosphate Port	Phosphate transportation	Al-Rousan et al. (2007)

### 2.2 Sample collection

Coral species samples (*S. pistillata*) were collected using SCUBA from the sampling sites of the GOA at a depth of 50 m. Coral mucus samples (*n* = 6) from each site were stored in sterile plastic bags and kept on ice during transport to the lab. The replicates were selected randomly from a 50 m transect area.

### 2.3 Mucus extraction

Coral mucus from the collected samples was extracted as described by Koren and Rosenberg (2006). Briefly, the samples were divided into small pieces and put in sterilized special tubes (50 ml) and then centrifuged (20 min at 4000 rpm). Subsequently, the samples were taken away and put in Eppendorf tubes (2 ml) to be stored at -20 °C.

### 2.4 DNA extraction, 16S library construction, and pyrosequencing

Total DNA was isolated from the collected mucus samples using a Genomic DNA purification kit (Promega, USA) following the manufacturer's instructions. The PCR amplification was done using primers for the V1–V3 region of the 16S rRNA gene. HotStarTaq Plus Master Mix Kit (Qiagen, Valencia, CA) was used to perform the amplification in 30 cycles with a single-step. The conditions used were as follows: 94 °C for 3 min, followed by 28 cycles of 94 °C for 30 s; 53 °C for 40 s and 72 °C for 1 min; and the last step was 72 °C for 5 min. Beads called Agencourt Ampure (Agencourt Bioscience Corporation, MA, USA) were employed to purify all amplicon products after mixing in even concentrations. Samples were sequenced using Roche 454 FLX according to manufacturer's guidelines as described by Dowd et al. (2008), by MR DNA, Shallowater, TX (www.mrdnalab.com).

### 2.5 Data analysis

A proprietary analysis pipeline (www.mrdnalab.com, MR DNA, Shallowater, TX) was employed to precede the sequencing of raw data. In summary, sequences were joined, depleted of barcodes, sequences <200 bp removed, and sequences with ambiguous base calls removed. Sequences were denoised, operational taxonomic units (OTUs) generated and chimeras removed. The OTUs were defined by clustering at 3% divergence (97% similarity); data were processed using (QIIME) software (Version: 1.7.1). Curated databases derived from Green-Genes, RDPII, and NCBI (www.ncbi.nlm.nih.gov, http://rdp.cme.msu.edu) were used along with BLASTn to obtain taxonomically classified final OTUs and compiled into each taxonomic level in both “counts” and “percentage” files.

Three measures, namely, Chao 1 richness (Chao, 1984), Shannon and Simpson indices, and a number of distinct OTUs were computed to evaluate diversity values in samples (α-Diversity). The total phylogenetic distance between the communities has been estimated using Fast UniFrac (Hamady et al., 2010) and visualized using UPGMA cluster created by PRIMER5 (Primer-E,UK).

## 3 Results

The sums of 39445 sequence reads were acquired as raw data from 454- pyrosequencing. After trimming the noise sequence of <200 bp, about 30345 (70%) sequence reads met the criteria and were analysed. Table 2 shows the number of sequence reads and the number of OTUs per sample. Site AQ4 represented the highest number of reads among all sites whereas the highest number of OTUs was obtained in the control site AQ1 (153 OTUs). AQ1 site showed the higher species diversity represented by the Shannon index value, which also showed the lowest value for the polluted site AQ2. The site AQ4 showed the highest community diversity measured by Simpson evenness (0.93), whereas the lowest values where measured for the AQ2 site. The Shannon index values were comparable (range: 1.68–3.37) across sampling sites indicating that the diversity of bacteria in the community is significantly different between the sampling sites affected by different pollution sources.

**Table 2:** Number of sequences, number of OTUs and α- diversity indices for different sampling sites.

	AQ1	AQ2	AQ3	AQ4	AQ5
No. of seq.	7,984	6,130	3,208	10,199	2,824
OTUs	153	105	85	80	64
Shannon	3.37	1.68	2.86	3.07	2.98
Simpson	0.91	0.49	0.88	0.93	0.92
Chao-1	158	115	91	98	73

**Fig. 2:**
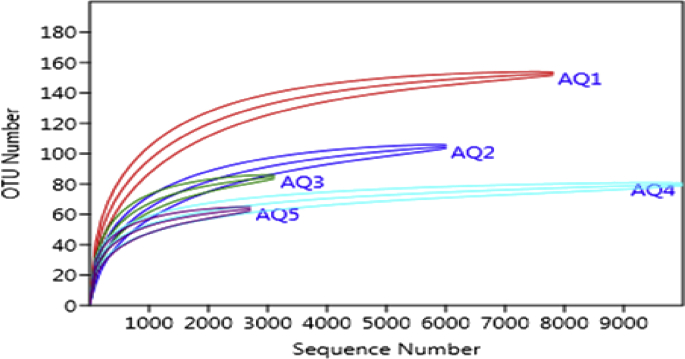
Rarefaction curve of 16S rDNA sequence reads obtained by pyrosequencing from coral associated bacteria from sampling sites. All sequences for each sample were considered for analysis.

Rarefaction curves (Fig. 2) suggested that the libraries in the samples included in this study were sufficient to capture most of the bacterial diversity. Remarkably the rarefaction curves showed higher OTU numbers in the AQ1 and AQ4 sampling sites than in the other samples, and also their were lower numbers in the AQ3 and AQ5 sampling sites. The control site AQ1 approached a plateau using the sequence reads.

### 3.1 Diversity and composition of bacterial communities associated with coral mucus

**Fig. 3:**
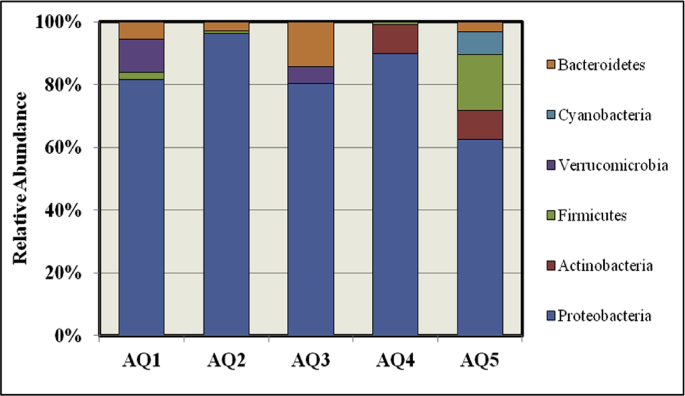
Taxonomic classification of bacterial reads from pooled DNA amplicons from various locations into phylum level using (QIIME) (50% confidence thresholds were applied for classification).

Average relative abundance in each sample of coral mucus showed the bacterial diversity represented at the phylum level (Fig. 3). Proteobacteria were found to be the predominant phylum in all sites. Sites AQ2 and AQ3 showed the least number of phyla. Furthermore, Cyanobacteria were detected only in site AQ5. Virrucomicrobia was a distinctive phylum in sites AQ1 and AQ3. Moreover, Actinobacteria was detected in sites AQ4 and AQ5, whereas; the relative abundance of Firmicutes was higher at AQ5. Additionally, the phylum Bacteroidetes was detected in all sites except AQ4.

**Fig. 4:**
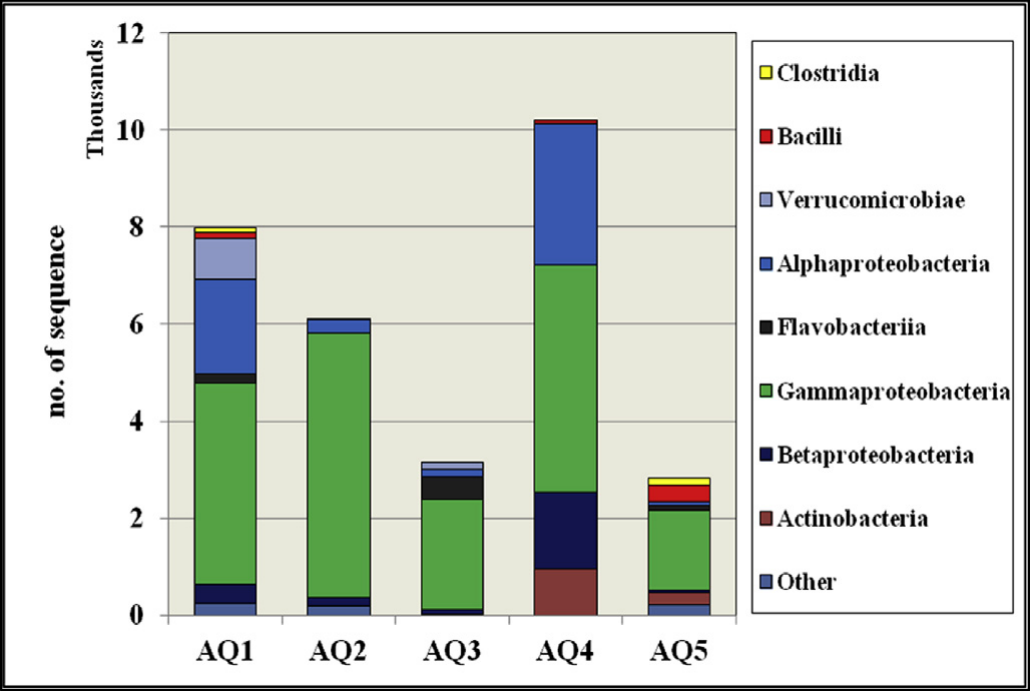
Taxonomic classification of bacterial reads obtained by using (QIIME) from pooled DNA amplicons into class level from various sites (50% confidence threshold was applied for classification).

The distribution of bacterial classes among all sampling sites is shown in Fig. 4. The highest number of reads among all sampling sites belonged to the class Gammaproteobacteria. The reads belong to Alphaproteobacteria, and Gammaproteobacteria were accounted for an average of 76% of the total reads among sampling sites. The highest percent (of Gammaproteobacteria) were obtained in the site AQ2 of 93 % of the total reads. The bacterial classes representing the lowest number of reads (donated by others) belonged to Cyanobacteria, Bacteroidia, Cytophagia, and Sphingobacteriia. These classes accounted for 3%, 3% and 7% of the sequences in the sites AQ1, AQ2, and AQ5 respectively. Verrucomicrobiae class was specifically detected in AQ1 and AQ3 sampling sites and accounted for 11 and 5 percent of the total number of reads for the respective site.

The dominant phylotypes of the coral mucus associated bacteria were members of the Gamma-, Beta-, and Alphaproteobacteria within the Proteobacteria (Table 3). A total of 92.25% of the total reads from AQ2 belonged to the sequence of Proteobacteria members, where as 60% of the sequences from AQ5 belonged to the Proteobacteria. The sequences belonging to the order Rhodobacterales were mostly dominant from class Alphaproteobacteria at AQ1 and AQ4. Burkholderiales of the Betaproteobacteria class were mostly dominant at AQ4. Within the Gammaproteobacteria class, Oceanospirillales were particularly abundant in all sampling sites except AQ4. A considerable increase in the number of sequences belonging to the order Vibrionales were observed at AQ4.

**Table 3:** Distribution of Proteobacteria taxa at different sampling sites.

Taxon	Classification	AQ1	AQ2	AQ3	AQ4	AQ5
Alpha	Rhizobiales	55	87	32	84	56
	Rickettsiales	107	0	54	0	0
	Rhodospirillales	12	0	0	804	0
	Sphingomonadales	20	41	10	0	10
	Rhodobacterales	1708	133	56	2008	0
Beta	Burkholderiales	181	138	82	1542	45
	Rhodocyclales	0	21	0	19	0
Gamma	Alteromonadales	166	6	65	0	0
	Oceanospirillales	3140	5177	1695	0	318
	Vibrionales	520	132	381	2939	293
	Pseudomonadales	117	58	29	799	1009
	Xanthomonadales	181	8	6	953	35

The relative abundance of the dominant OTUs between sampling sites was highly variable. For instance, OTU1, which belonged to Endozoicomonas sp. were accounted for by 71.2 % of the total abundance at AQ2, and 27, 26 % of the total abundance at AQ1 and AQ3, respectively. Interestingly, this OTU wasn't present at the site AQ4. The OTU2 which belong to the pathogenic bacteria *Vibrio ponticus* were presented in varying abundances at all sampling sites, with the highest abundance of 22.9% of the total reads at AQ5 sampling site. Surprisingly, the OTU 2, 25, 87, 180 and 231 shared between all sampling sites, belonged to the genus *Vibrio*.

### 3.2 Similarity among bacterial communities

**Fig. 5:**
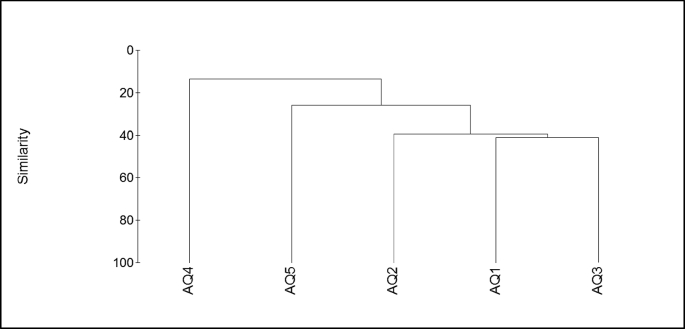
UniFrac UPGMA cluster of bacterial communities associated with coral mucus from different sampling sites.

A UPGMA hierarchical clustering was calculated to compare the similarities between different coral mucus microbial communities from various ecological sites (Fig. 5). The cluster created was based on each OTU ′s relative abundance to calculate the similarity between samples using the Bray-Curtis dissimilarity distance.

The bacteria associated with coral *S. pistillata* mucus from the AQ1 and AQ3 sites generally clustered together, which indicates that they shared high similarities with each other. This cluster was well separated from another cluster formed by the samples from sites AQ2, AQ4, and AQ5, suggesting a substantial dissimilarity of the bacterial communities in these samples compared with those associated with site AQ4 and AQ5. The bacterial communities associated with coral mucus at site AQ4 did not share any similarity in terms of the composition of associated microbes form distinct clusters.

The similarity among mucus-associated bacterial communities was also measured using a test of similarity (SIMPER). The average dissimilarity between the control site (AQ1) and the polluted sites were 68.1%, whereas, the average similarity percent between polluted sampling sites (AQ2, AQ3, AQ4, AQ5) was 25.4%.

## 4 Discussion

In this study, the microbial structure associated with coral mucus was analyzed from samples collected at five sampling sites on the Jordanian coast of the GOA using bTEFAP approach. To our knowledge, this is one of the first studies to characterize coral associated bacterial community at the GOA area using a new generation sequencing technique (NGS).

Culture-independent results using the bTEFAP approach revealed a higher diversity of associated bacterial communities with corals than were previously reported using clone libraries or DGGE community profiling (Jacob et al., 2017; Yakimov et al., 2006). The results obtained from this study were consistence with these findings of higher community diversity and abundance compared to the results obtained by Zhang et al. (2015). The values of alpha diversity indices for our study was consistence with the study of Zhang et al. (2015) where they declared that community diversity was higher for an associated coral community using culture-free, 16S rRNA based techniques including DGGE community profiling at Luhuitou fringing reef, China (Shannon index 2.15–2.71; Simpson evenness 0.16–0.38), than what was previously reported with culture-based techniques.

Most sequence readings in each sampling site were accounted for by a small number of OTUs at all sites, and this shows that there is a core bacterial community associated with mucus in all the study areas. These OTUs were categorized as alpha-, beta- and gamma- Proteobacteria, Bacilli, Flavobacteriia, and Verrucomicrobia, which were described as ubiquitous species from healthy and diseased coral using the clone-based library and pyrosequencing approaches (Cárdenas et al., 2012). Changes in community composition from one site to another were probably due to changes in the presence and quantities of less abundant OTUs and changes in the abundance of dominant genera. Interestingly, 82% of identified OTUs could be assigned to species of the Proteobacteria phylum. These results were in agreement with those of Telfah (2012); Jaber (2012), where they found that Proteobacteria was the predominant phyla in the mucus of *S. pistillata* samples in the same study area based on culture dependent methods. On the other hand, an average of 8 % of the total sequence reads was assigned for eight non-ubiquitous classes (i.e., Actinobacteria, Cyanobacteria, Bacteroidia, Cytophagia, Deinococci, Sphingobacteriia, and Clostridia). It can be postulated that these classes of bacteria belong to the 'rare biosphere', which makes them able to explain differences in community structure from one site to another, as indicated previously (Sogin et al., 2006).

The study of associated microbial communities over different pollution sources at the GOA revealed an influence of the anthropogenic pollutants on the coral *S. pistillata* mucus associated microbial communities. This is in agreement with the hypothesis that disturbances in the dynamic equilibrium of the coral microbiome may result in health deterioration (Lesser et al., 2007). Previous studies revealed the fact that the structure of the microbial community is also determined by environmental factors as well as physiological conditions of the coral holobiont (Hong et al., 2009).

The highest number of OTUs (as indicated by the rarefaction curve, Fig. 2) was obtained for AQ1, which was selected as an uncontaminated site. The increased swimming and touristic activities beside the solid waste discharge at AQ4 resulted in the highest bacterial diversity among sites; this was clearly shown by the similarity obtained by UPGMA hierarchical clustering. This can be described by deterioration caused by stress of the host-microbial balance, leading to some uncontrolled appearance of irregular and harmful taxa (Rosenberg and Kushmaro, 2011). For instance, a high prevalence of the pathogenic bacteria from the order Vibrionales at site AQ4 can be related to the increased disturbances and coral mucus aging as discussed by Glasl et al. (2016). Host-species specificity decreased at the contaminated sites (AQ2, AQ3, and AQ5). However, loss of species specificity and higher similarity of coral microbial formations in corals suffering from contamination have been demonstrated previously (Frias-Lopez et al., 2002; Cárdenas et al., 2011). Pollutants seem to negatively influence the balance between the principal microbial taxa that are linked to coral mucus. This trend has also been observed in the present study by the decrease in the bacteria belonging to the Hahellaceae family (species Endozoicomonas), Hydrogenophilaceae and Verrucomicrobiaceae. This corresponds to the microbial response due to stress in the other species and emphasizes the significance of the rare (opportunistic) bacterial biosphere, as indicated previously (Sunagawa et al., 2010; Jessen et al., 2013). The compositional shift in the microbial communities of the stressed sites at the GAO exhibited an increased relative abundance of bacterial families such as Verrucomicrobiaceae, Oceanospirillaceae, and Flavobacteriaceae. These findings are consistent with a previous study attributing the expression of lesions in *Porites astreoides* colonies to the loss of Endozoicimonaceae and the proliferation of an opportunistic bacterial community (Meyer et al., 2014). Furthermore, the coral mucus suffered from different pollution sources that exhibited increased relative abundances of bacterial families described as early colonizers of marine biofilms, such as Rhodobacteraceae and Oceanospirillacae, which was in agreement with the results obtained by O'Toole et al. (2000).

The diversity of microbial communities (as indicated by No. of OTUs, Table 2) was generally reduced at polluted sites as compared to the uncontaminated site. These findings do not agree with the observations of Jessen et al. (2013), where exposure of *Acropora hemprichii* to simulated Red Sea stressors exhibited by overfeeding and excessive fishing has been shown to augment microbial diversity with time passing, notwithstanding lack of experimental control in research design. Moreover, the prevalence of diverse bacterial species also seems to be a typical characteristic of diseased coral, as shown, for instance, in White Plague stressed corals *Diploria strigosa* and *Siderastrea siderea* (Cárdenas et al., 2011). This was obvious for the increased abundance of pathogenic species of the family Vibrionaceae and Pseudomonadaceae, especially at sites AQ4 and AQ5.

The bacterial communities was characterized by the presence of several abundant nutrient cycling OTUs. This can be related to higher levels of nutrients in the polluted sampling areas. Hence, species of the genus *Vibrio* are reported to use sulfur compounds produced by coral as a cue to target stressed corals (Garren and Azam, 2010). Some OTUs belonged to genera of the order Rhizobiales (Bradyrhizobium lupine), and Pseudomonadales (pseudomonas sp.) at the sites AQ4 and AQ5 subjected to sedimentation and local sewage resulting from the extensive industrial (phosphate transportation at AQ5 site) and solid waste disposal, and tourist activities (AQ4). Moreover, these OTUs include taxa that are potentially involved in cycling nitrogen (Lema et al., 2012). Furthermore, some OTUs (i.e. OTU3) belonged to the human skin microbiome, mainly skin topographical and temporal forearm clones (Kong et al., 2012) presented at AQ3, AQ4, and AQ5 represented by anthropogenic influences at these sites.

The microbial community at site AQ1 contains OTUs of the order Oceanospirillales (*Alcanivorax* sp.) and Novosphingobium (Sphingobacteriia) that have been found in environments contaminated by crude oil (Tang et al., 2012; Wang et al., 2014) and contribute to the quality deterioration of sulfur compounds like dimethyl sulfoniopropionate (González et al., 2003). These OTUs can be related to accidental oil spills from the adjacent area or result from boating activities.

The shift of coral mucus associated with microbial community structure in the sampling sites is affected by pollution sources and can be interpreted as coral holobiont acclimatization methods following changes in environmental statuses that enhance the probiotic hypothesis (Reshef et al., 2006). Nevertheless, the changes pose a threat to coral holobionts in related microbial structure, in which the non-stop exposure to stressors is not yet visible (Rosenberg and Kushmaro, 2011). The shift of microbial communities under local pollution sources may represent a shift in favor of the disease due to loss of microbial communities specific to coral host or the increase in alpha diversity (Ziegler et al., 2016).

## 5 Conclusion

In summary, the mucus of the coral *S. pistillata* from the GOA sampling sites exhibits a high diversity of associated bacterial communities. The pollution sources in different sites along the GOA affected the coral-associated holobiont, resulting in a shift of microbial communities due to increased human activity. Additionally, pyrosequencing techniques proved to be an excellent tool for the study of microbial community shifts in the marine environment. Further studies are required of coral reef-associated microbial communities in the GOA to investigate the microbial effect on coral health.

## Declarations

### Author contribution statement

Emad Hussien: Conceived and designed the experiments; Analyzed and interpreted the data; Contributed reagents, materials, analysis tools or data; Wrote the paper.

Abdul-Salam Juhmani, Ruba AlMasri: Performed the experiments.

Fuad Al-Horani: Performed the experiments; Analyzed and interpreted the data.

Mohannad Al-Saghir: Analyzed and interpreted the data; Wrote the paper.

### Funding statement

This work was supported by Yarmouk University.

### Competing interest statement

The authors declare no conflict of interest.

### Additional information

No additional information is available for this paper.
